# Influence of Nutriment on Disease

**Published:** 1869-11

**Authors:** 


### Influence of Nutriment on Disease.

The *Journal Central de Medicine de Berlin* publishes *seriatim* the conclusions arrived at by Dr. Salisbury from a large number of experiments on this subject. They are as follows:—

1.—The continuous use of vegetable aliments, particularly of the leguminous and amylaceous plants, provokes constipation and a sort of scorbutic condition.

2.—The use of amylaceous plants causes a great variety of morbid conditions; for example, fibrinous deposits and embiola in the capillary vessels, and congestion, inflammation, diarrhœa and paralysis, as the result, and, at a later period, tuberculous deposits in the lungs, affections of the eyes and ears, and pains in the back and extremities.

3.—These symptoms may be advantageously met by having recourse to albuminoid ailments of animal origin, and especially by the use of salts of potash and iron, which prevent coagulation of fibrin, favor the circulation of the blood, and render active absorption and intestinal secretion.

4.—The morbid states which we have enumerated are developed in armies on the field, especially when they are subjected to an amylaceous dietary.

5.—The officers, who have the privilege of varying their diet, are free from these accidents.

6.—When the nutriment is exclusively amylaceous or saccharine, constipation soon appears, and stomachic digestion is effected with difficulty.

7.—This constipation is not slow in engendering the products of fermentation, intestinal gases, and parasites in great number.

8.—This condition continues until diarrhœa sets in, and then small colloid gelatinous masses are found in the fæces in variable quantity.

9.—These are in no respect the cause of the diarrhœa; they result from the production and metamorphosis of saccharine and fermentative matter. Once produced, these substances act as a true poison on the organism and aggravate the intestinal lesions.

10.—It is specially in the evening and during the night that the products of fermentation are developed in the intestines. Each day they increase. The continual excitement of the intestinal mucous membrane by the gases and parasites produces diarrhœa, which soon becomes chronic, and resists all method of treatment.

11.—This state is always accompanied with a tendency to paralysis accompanied by singing in the ears and violent headache, symptoms which are attributable to defective nourishment and to the presence of fibrinous masses in the capillary vessels.

12.—There are often at this stage symptoms of bronchitis. The expectoration is thick, yellow, principally at night, and it is complicated with dyspnœa and palpitations.

13.—Diabetes and strumous affections often supervene on a diet exclusively saccharine or containing too much starch.

14.—The diarrhœa, characterized by colloid greenish stools, which attacks children during the summer, depends on the abuse of saccharine substances and fruits, and the colloid masses are derived from the intestinal epithelium.—*Medical Record.*

